# SSELM-neg: spherical search-based extreme learning machine for drug–target interaction prediction

**DOI:** 10.1186/s12859-023-05153-y

**Published:** 2023-02-03

**Authors:** Lingzhi Hu, Chengzhou Fu, Zhonglu Ren, Yongming Cai, Jin Yang, Siwen Xu, Wenhua Xu, Deyu Tang

**Affiliations:** 1 School of Medical Information Engineering, Guangdong Pharmaceutical University, Guangzhou, People’s Republic of China; 2 School of Computer Science and Engineering, South China University of Technology, Guangzhou, People’s Republic of China; 3 Guangdong Province Precise Medicine Big Data of Traditional Chinese Medicine Engineering Technology Research Center, Guangzhou, People’s Republic of China

**Keywords:** Drug–target interactions, Drug discovery, Extreme learning machine, Spherical search, Class imbalance

## Abstract

**Background:**

The experimental verification of a drug discovery process is expensive and time-consuming. Therefore, efficiently and effectively identifying drug–target interactions (DTIs) has been the focus of research. At present, many machine learning algorithms are used for predicting DTIs. The key idea is to train the classifier using an existing DTI to predict a new or unknown DTI. However, there are various challenges, such as class imbalance and the parameter optimization of many classifiers, that need to be solved before an optimal DTI model is developed.

**Methods:**

In this study, we propose a framework called SSELM-neg for DTI prediction, in which we use a screening approach to choose high-quality negative samples and a spherical search approach to optimize the parameters of the extreme learning machine.

**Results:**

The results demonstrated that the proposed technique outperformed other state-of-the-art methods in 10-fold cross-validation experiments in terms of the area under the receiver operating characteristic curve (0.986, 0.993, 0.988, and 0.969) and AUPR (0.982, 0.991, 0.982, and 0.946) for the enzyme dataset, G-protein coupled receptor dataset, ion channel dataset, and nuclear receptor dataset, respectively.

**Conclusion:**

The screening approach produced high-quality negative samples with the same number of positive samples, which solved the class imbalance problem. We optimized an extreme learning machine using a spherical search approach to identify DTIs. Therefore, our models performed better than other state-of-the-art methods.

## Introduction

Drug–target interaction (DTI) prediction is an important way to reposition drugs [1–4] that not only plays a crucial role in the development of new drugs [5] but is also essential for studying the adverse reactions of drugs [6, 7]. However, it is time-consuming and expensive to verify DTIs using wet experimental methods [8, 9]. An important issue is how to reduce the cost of drug development. Thus, the in silico approach becomes essential because it improves the accuracy of finding drug–target relationships and saves time [10]. With the increasing number of public databases [11], different computational strategies can be more effectively applied for DTI prediction [12].

Generally, computational methods for DTI identification can be divided into three categories: ligand-based methods [13], docking-based methods [13], and chemogenomic methods [14]. These methods have played an important role in predicting DTIs; however, docking methods that use the three-dimensional (3D) structures of drugs and proteins, and then perform simulations to determine whether they interact, because of the limited 3D crystal structure of known targets, so there are limitations [15–17]. Ligand-based methods are based on the fact that similar molecules tend to have similar properties and usually bind similar proteins [18], which means that when the number of known ligands per protein is insufficient, the prediction results of ligand-based methods may become unreliable [19].

Chemogenomic methods use both drug and target information to integrate the chemical space of the drug and the protein space of the target into a pharmacological space to predict DTIs. An advantage of chemogenomic approaches is that they can work with widely abundant biological data to perform prediction [14]. We classify the chemogenomic methods into four types: machine learning methods, matrix factorization methods, network-based methods, and hybrid methods.

There are three branches of machine learning methods for predicting DTIs: similarity-based methods, deep learning methods, and feature selection methods. Similarity/distance-based methods mainly use inter-sample similarity or distance [20–22]. Yamanishi et al. [23] developed a bipartite graph model to predict DTIs using a supervised approach to learn known drug–target relationships [24, 25]. Buza et al. proposed ECkNN/HLM, which is a *K*-nearest neighbor (KNN) method (hub-aware regression technique) with error correction, to mitigate the harmful effects of bad hubs [26–28]. Mei et al. proposed BLM-NII, which is an inference integrated into a BLM approach, to solve a new candidate problem for pure BLM [29]. However, the main disadvantage of this set of methods is that only a few drugs and their interactions are known, and there is a large amount of unlabeled data in the dataset [30]. The application of deep learning methods in drug discovery has been increasing because of their excellent performance [31, 32]. Wen et al. proposed DeepDTIs, using DBN [33] to extract raw input vectors and predict new DTIs between FDA-approved drugs and targets [34]. Lee et al. proposed DeepConv-DTI, which is a deep learning method, to obtain local residue patterns of proteins involved in DTI [35]. You et al. proposed LASSO-DNN, which is a deep learning method based on features extracted from LASSO regression models using protein-specific features and drug-specific features for fitting [36]. The disadvantage of such methods is how to select truly non-interacting drug–target pairs [37]. Feature-based methods are currently the vast majority of machine learning methods that perform DTI prediction. They comprise a broad range of methods, including the support vector machine (SVM), tree-based methods, and other kernel-based methods. SVM, KSVM, MH-SVM, and other methods have been proposed. The main principle is that an SVM constructs one or a set of hyperplanes, which can be used to predict whether there is an interaction between a drug and target [19, 38–42]. Xia et al. proposed NetLapRLS, which is an improved version of LapRLS, by incorporating new kernels built from known DTI networks [43]. However, the problem encountered by such methods is that the lack of 3D structure of membrane proteins hinders the extraction of key features.

Matrix factorization methods have achieved better results in DTI prediction. GRMF-WGRMF is a two-manifold learner for extracting low-dimensional nonlinear manifolds of DTI bipartite graphs proposed by Ezzat et al. [44]. Gönen et al. proposed a method to decompose the interaction score matrix into a kernel matrix (similarity matrix), which can be used as DTI predictors for the new drug and protein KBMF2K [45]. The disadvantage of this type of approach is that the rapid growth in the amount and variety of data related to a drug and/or target far exceeds the capabilities of matrix-based data representations and many current analysis algorithms. The network-based approach uses graph-based techniques to perform DTI prediction, which has the advantage of being simple and reliable. Luo et al. proposed DTINet, which is a computational network integration pipeline for DTI prediction [46]. Chen et al. proposed NRRRH, which is a latent DTI inference method for bipartite graph networks based on the random walk with restart (RWR) framework [47]. The RWR proposed by Seal et al. is a method that requires matrix inversion and provides a good correlation score between two nodes in a DTI-weighted graph [48].

Hybrid methods refer to all methods that use any combination of feature-based methods, matrix factorization, deep learning, and network-based methods. Domain tuned-hybrid proposed by Alaimo et al. is an extended NBI technique that combines domain-based knowledge, such as drug similarity and target similarity [49]. By reviewing the above methods, we found that a key problem is how to select negative samples; hence, the first problem we solve in this study is to establish a highly reliable negative sample dataset to overcome the shortcomings of previous methods. The number of negative samples is much larger than the number of positive samples. There will be a class imbalance problem, which will affect the prediction accuracy of the final DTI. Therefore, in this study, we choose a screening method to build a highly reliable negative sample dataset to solve the class imbalance problem.

Additionally, we propose a new classifier for predicting DTIs: an extreme learning machine (ELM) based on spherical search (SS) optimization. An ELM is a popular machine learning method that has been widely applied to real-world problems because of its fast training speed and good generalization performance [50]. Previously, scholars have used an ELM to predict the new relationship between drugs and targets. However, the network parameters are randomly generated, which reduces the prediction performance of the ELM model. Therefore, using the swarm intelligence algorithm to optimize the network parameters of the ELM is necessary. SS is a swarm intelligence algorithm that has few adjustment parameters; its accuracy, convergence rate, proficiency, and effectiveness are at an advanced level; and it has projection characteristics, which can eliminate stagnation during the search process, which is conducive to eliminating sticky in local minima.

Therefore, we propose a framework called SSELM-neg for predicting the DTI. The innovations in this study are as follows:

1. We propose a DTI prediction framework using the screening approach and SS-based ELM.
2. We form a high-confidence negative sample dataset using a screening approach based on the principle that dissimilarity between a new drug and a drug with a known (predicted) protein precludes its possible correlation with the protein.
3. We propose an SS-based ELM. We optimize the parameters of the ELM using SS to improve the classification performance of DTIs.

## Related work

In this study, we focus on machine learning methods to predict DTIs. Currently, this has three main problems: (1) complexity of training sample generation; (2) generation of credible negative samples; and (3) performance of the classifier.

The method used to train sample generation for machine learning is divided into a raw data generation method (feature-based method) and data integrated method using similarity scores (similarity-based method). The feature-based method requires feature selection; hence, it requires the drug–target pairs to be explicitly represented as fixed-length feature vectors, which can lead to a large number of complex calculations. By contrast, similarity-based methods do not require feature extraction or selection and are simpler to compute than that. The principle of the similarity-based DTI prediction method is to generate the similarity matrix of drugs by calculating the chemical structure of drugs and the similarity matrix of targets by calculating the characteristic of proteins, and finally, these two similarity matrices are used in various classification methods, such as [51].

However, whether feature-based methods or similarity-based methods are used to generate training sets, the number of negative samples far exceeds the number of positive samples because generally, unrecognized DTIs are considered as negative samples. This leads to data imbalance, which greatly reduces the accuracy of the classifier. The traditional method is to extract negative samples randomly. In recent years, some methods (not more) for extracting negative samples have been proposed. Mohammad et al. proposed the BRNS algorithm to extract balanced and reliable negative samples [52]. Jiaying You et al. [53] proposed a novel method to select the most likely negative DTIs. The assumption of this method is based on “guilt-by-association,” which indicates that similar drugs may share similar targets and vice versa. However, these methods are often more complex to calculate. Therefore, in this study, we use a simpler screening method to extract a more credible negative sample based on the study of Liu et al. [42].

Additionally, the performance of the classifier is particularly important in machine learning-based methods for predicting DTIs, and more classical classifiers, such as SVMs [54–56], KNN [57, 58], and random forest [59, 60], have been used. The ELM has received a great amount of attention because of its excellent performance, and is also used in many areas [61–63], such as power and finance. Xin et al. [64] used ELMs for drug-drug interaction prediction, and An et al. [65] used kernel ELMs to identify DTIs based on drug fingerprints and protein evolutionary information. To date, few studies have been conducted in which researchers have used ELMs for the prediction of DTIs. One important reason is that the configuration of the hidden layer parameters of an ELM network requires better optimization methods.

## Preliminaries

ELM is an algorithm proposed by Huang et al. [66] for solving a single hidden layer feedforward neural network. It initializes and randomly generates input weights and hidden layer biases, and uses a nonlinear activation function to map the input data to the new feature space. Its advantages are that it can minimize the training error, obtain the smallest weight norm and best generalization performance, and the learning speed is fast.

The number of input samples is *N* and the samples are $(x_i,t_i)$, where $x_i=[x_{i1},x_{i2},\ldots ,x_{in}]^T \in R^n$ and $t_i=[t_{i1},t_{i2},\ldots ,t_{in}]^T \in R^m$. The weights of the output layer are represented by the generalized inverse of the output matrix of the hidden layer. Hence, the ELM is expressed as

1 $$\begin{aligned} \begin{aligned} t_j=\sum _{i=1}^{L}\beta _ig(x_iW_i+b_i), \qquad \qquad j=1,2,\ldots ,N, \end{aligned} \end{aligned}$$

where *L* is the number of hidden layer nodes, $w_i=[w_{i1},w_{i2},\ldots ,w_{in} ]^T$ is the weight vector that connects the input layer and hidden layer, $b_i=[b_{i1},b_{i2},\ldots ,b_{in}]^T$ is the bias vector of the hidden layer, and $\beta _i=[\beta _{i1},\beta _{i2},\ldots ,\beta _{im}]^T$ is the weight vector that connects the hidden layer and output layer. $G(x)=[g(x,w_1,b_1),g(x,w_2,b_2),\ldots ,g(x,w_n,b_n )]$ represents the activation of the hidden layer function.

The learning goal of the single hidden layer neural network is to minimize the error of the output. When the error between the output result and sample *N* is zero, the above formula can be abbreviated as

2 $$\begin{aligned} \begin{aligned} H\beta =T, \end{aligned} \end{aligned}$$

where

3 $$\begin{aligned}{} & {} H={ \left[ \begin{array}{c} h(x_1) \\ \vdots \\ h(x_N) \end{array} \right] }={ \left[ \begin{array}{ccc} h_1(x_1) &{} \cdots &{} h_L(x_1) \\ \vdots &{} \cdots &{} \vdots \\ h_1(x_N) &{} \cdots &{} h_L(x_N) \end{array} \right] } \end{aligned}$$

4 $$\begin{aligned}{} & {} T={ \left[ \begin{array}{c} t_1^T \\ \vdots \\ t_n^T \end{array} \right] }, \end{aligned}$$

where *H* is the output of the hidden layer node, $\beta$ is the output weight, and *T* is the expected output. After applying the Moore–Penrose generalized inverse operation, we obtain

5 $$\begin{aligned} \begin{aligned} \beta =H^\dag T, \end{aligned} \end{aligned}$$

where $H^\dag$ is the generalized inverse of matrix *H*.

## Methods

### Problem description

Our problem is based on the assumption that there is a drug set $D \in \{d_1,d_2,\ldots ,d_n\}$ and protein set $P \in \{p_1,p_2,\ldots ,p_n\}$, where *D* contains *n* drugs and *P* contains *m* proteins. The relationship between the drug and target protein is defined as an $m\times n$ binary matrix *Y*, where the drug interacts with protein $p_j$, $y_{ij}=1$; when drug $d_i$ does not interact with target protein $p_j$; or the interaction is unknown. The similarity between drugs is represented by matrix $S_d$ and the similarity between target proteins is represented by matrix $S_P$. We calculated the prediction scores for each non-interacting drug–target pair and predicted new drug–target pairs.

### Construct the negative sample set

**Fig. 1:**
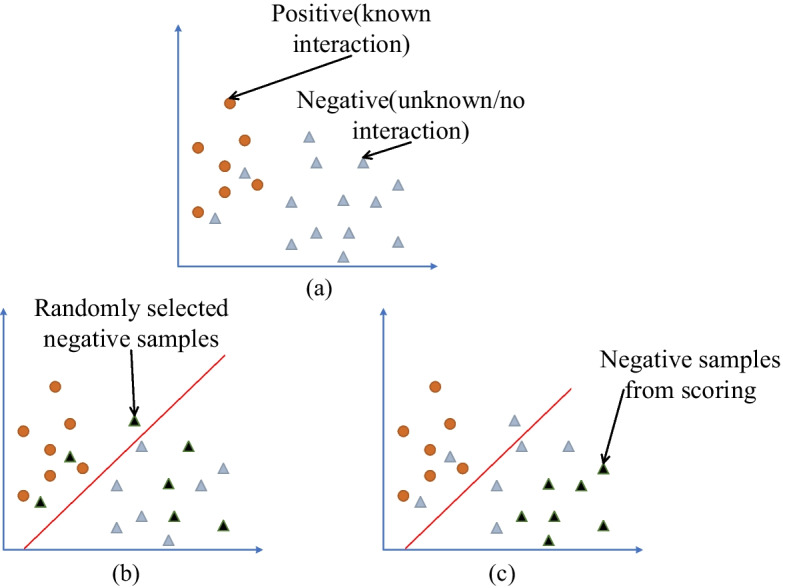
Visual of the negative samples selected

The number of negative samples (unverified samples) in the drug–targeted interaction dataset was significantly higher than the number of positive samples (verified samples, as shown in Fig. 1a), which resulted in a decrease of the predictive performance of classification for drug–targeted interaction because of the data imbalance. To balance the dataset, in previous studies, researchers frequently used random selection methods to extract negative samples that were consistent with the size of the positive samples, as shown in Fig. 1b. However, this overlooks a critical issue: unlabeled DTIs may have interactions that have not been discovered or argued for. The random selection of negative samples may result in choosing some unlabeled DTI samples as negative samples; however, they are probably positive samples, which reduces the performance of the model. The proposed screening approach is to extract high-quality negative samples. (These negative samples are far away from the positive samples, as shown in Fig. 1c.) We set all known DTI labels to 1 and all other chosen samples in the DTI space (drug–target pairs with no known interactions) to 0. We directly include all samples with labels of 1 in the dataset as positive samples and use all samples with labels of 0 as negative samples.

Building the assembly *K* of the known/predicted DTIs as mentioned above, and using the protein dissimilarity rule and drug dissimilarity rule [42], we integrate similarities between drugs into a drug composite similarity score, as is the case for similarities between proteins. This can be represented by $(c_k,p_j,d_{kj})$, where $c_k$ represents drug *k*, $p_j$ represents protein *j*, and $d_{kj}$ represents the interaction between drug $c_k$ and protein $p_j$. For any protein $p_l$ targeted by $c_k$ in *K*, we compute the weighted score $spc_{jkl}=w_{kl}*PS_{jl}$ that indicates the possibility that protein $p_j$ and each known/predicted protein $p_l$ are targeted by drug $c_k$, that is, $(c_k,p_l,w_{kl} \in K)$. We calculate the combined score by summing the weighted scores $spc_{jkl}$ with respect to *l* and thus obtain

6 $$\begin{aligned} \begin{aligned} SPC_{jk}=\dfrac{\sum _{l}p_{jk}\times spc_{jkl}}{\sum _{l}spc_{jkl}}. \end{aligned} \end{aligned}$$

Similarly, we compute the weighted score $scp_{kj}=w_{ij}*CS_{ik}$ that represents the possibility that drug $c_k$ targets $p_j$ in consideration of the similarity between $c_k$ and each known/predicted drug $c_i$ that targets protein $p_j$, that is, $(c_i,p_j,w_{kl}) \in K$. We calculate the combined score by summing the weighted scores $spc_{kji}$ with respect to *i* and thus obtain

7 $$\begin{aligned} \begin{aligned} SCP_{kj}=\dfrac{\sum _{i}p_{kj}\times scp_{kji}}{\sum _{i}scp_{kji}}, \end{aligned} \end{aligned}$$

where $p_{kj}$ is the interaction value between protein $p_j$ and drug $c_k$, and$scp_{kji}$ is the similarity between drug $c_{k}$ and all others.

For target drug $c_k$ and protein $p_j$, the average weighted score is defined as

8 $$\begin{aligned} \begin{aligned} S_{kj}=\dfrac{SPC_{k}+SCP_{j}}{2}. \end{aligned} \end{aligned}$$

We choose the potential negative samples according to the sorted scores obtained from Eq. (8), and those with the lowest scores form the negative sample candidate set. We combine the positive samples and negative samples to obtain the train dataset and test dataset. We conducted the experiments in this study on this dataset. The dataset (DTI pairs) are represented as

9 $$\begin{aligned} \begin{aligned} DTI_{i, G}=[P_{i1},P_{i2},\ldots ,P_{in}; C_{j1},C_{j2},\ldots ,C_{jm};D_{i,j}], \end{aligned} \end{aligned}$$

where $C_i$ denotes the drug, $P_i$ denotes the protein, and $D_{ij}$ denotes the classification label (0 or 1) between drug $C_i$ and protein $P_i$.

In Fig. 1a, yellow circles represent known drug target pairs and gray triangles represent unknown or unrelated drug target pairs. The closer the gray triangles to the *y*-axis, the greater the likelihood of inter-relationships. Figure 1b shows the randomly selected negative samples, which are represented by black triangles. The black triangles close to the left of the red line probably have DTI and they are probably positive samples, but they were chosen as negative samples. Figure 1c shows the negative samples selected using the screening approach, and the black triangles are far from the red line.

### Extreme learning machine based on spherical search

The evolutionary algorithm is an optimization method that can be used to solve general optimization problems because it is simple and flexible, has no derivatives, and avoids falling into a local optimum [67]. The SS is an evolutionary algorithm for solving nonlinear bounded constrained global optimization problems [67]. To date, it has not been used to solve the parameter optimization problem of an ELM. In this study, we use it to optimize the network parameters of an ELM. First, we initialize the population of the SS algorithm using random selection. We define the population in the *G*th iteration as $Q_x$, which is expressed as

10 $$\begin{aligned} \begin{aligned} Q_{x, G}=[x_{1,G},x_{2,G},\ldots ,x_{i,G},\ldots ,x_{N,G}], \end{aligned} \end{aligned}$$

where $x_{i,G}$ is the solution in the population, $x_{ij}$ is the *j*th element of the *i*th solution, and $x_{ij}$ is a parameter of the ELM. $x_{i,G}$ is a vector in the *D*-dimensional search space. *D* denotes the number of parameters in the ELM.

**Initialization of the solution: ** Choose a random distribution between the upper and lower dimensions of the *j*th element to initialize the solution as

11 $$\begin{aligned} \begin{aligned} x_{ij,0}=(x_{uj} - x_{lj})\times rand(0,1]+x_{lj}, \end{aligned} \end{aligned}$$

where $x_{uj}$ and $x_{lj}$ are the upper and lower dimensional boundaries of the *j*th element, respectively. *rand*(0, 1] represents the generation of uniformly distributed random numbers in (0, 1].

**Generation of trial solutions: ** Trial solutions are new potential solutions generated through iteration and competition:

12 $$\begin{aligned} \begin{aligned} y_{i,G}=x_{i,G}+v_{i,G}\times m_{i,G} \times z_{i,G}, \end{aligned} \end{aligned}$$

where $m_{i,G}$ is a projection matrix that determines the value of $y_{i,G}$ on the $D-1$ dimensional spherical boundary; different $p_{i,G}$ result in different $y_{i,G}$ values:

13 $$\begin{aligned} \begin{aligned} m=A'diag(b)A, \end{aligned} \end{aligned}$$

where *A* is an orthogonal matrix,

14 $$\begin{aligned} \begin{aligned} AA'=I, \end{aligned} \end{aligned}$$

where *b* is a binary vector, and

15 $$\begin{aligned} \begin{aligned} 0<rank(diag(b_i))<1. \end{aligned} \end{aligned}$$

The position of $y_{i,G}$ determines the spherical boundary of dimension $D-1$, and $x_{i,G}$ is a specific solution. $c_{i,G}$ represents the step size control vector, which is randomly calculated in [0.5, 0.7].

$z_{i,G}$ represents the search direction. In optimization algorithms, the quality of new solutions is highly dependent on the balance between the exploration and utilization of the search space. We use two search operations: $towards-best$ and $towards-rand$. We use the $towards-rand$ method in the half of the population with a better solution because it has a better search ability, and use the $towards-best$ method in the other half because it has a better search ability. The combination of the two search directions provides a balance for the exploration and utilization of the search space, which not only improves the diversity of better solutions but also forces poor solutions to improve fitness:

16 $$\begin{aligned}{} & {} {\textbf {towards-rand}} \quad z_{i,G}=x_{pi,G}+x_{qi,G}-r_{ri,G}-x_{i,G} \end{aligned}$$

17 $$\begin{aligned}{} & {} {\textbf {towards-best}} \quad z_{i,G}=x_{pbesti,G}+x_{qi,G}-r_{ri,G}-x_{i,G}, \end{aligned}$$

where $p_i$, $q_i$, and $r_i$ are the index numbers randomly selected from 1 to *N*, and $x_{pbesti,G}$ is a randomly selected individual using the top *p* optimal solutions. $x_{pbesti,G}$ and $x_{pi,G}$ represent target points. $(x_q-r_r)$ is the difference term, and $x_q$ and $r_r$ are randomly selected individuals from the current solution set; hence, the actual search direction may deviate from the target search direction, to a certain extent.

We use Success History-based-control Parameter Adaptation (SHPA) [68] to adapt two control parameters during the search: rank and $c_i$. SHPA creates a history matrix *L* of size $(2 \times H)$ to hold *H* entries for the two control parameters, that is, the learning values $l_r$ and $l_c$ for parameters *rank* and *c*, respectively, in the last *H* iterations.

$rank_{i,g}$ and $c_{i,g}$ are calculated as

18 $$\begin{aligned}{} & {} rank_{i,g}=Binornd(D,L_{1,j}) \end{aligned}$$

19 $$\begin{aligned}{} & {} c_{i,g}=Cauchyrand(L_{(2,j},0.1), \end{aligned}$$

where Binornd represents the binomial distribution, *j* is chosen independently from the columns of matrix *L*, and each *i* is random. Cauchyrand represents the Cauchy distribution, *j* is chosen independently from the columns of matrix *L*, and each *i* is random.

The performance of SS is highly dependent on the control parameters $c_i$, and the rank and size of population *N* [67]. In this study, we use the exponential population size reduction method to dynamically adjust the population size during the iterative process. We exponentially reduce the population as a function of the number of iterations by continuously reducing the population to match the exponential function. The population size is $N_{init}$ at the first iteration and $N_{min}$ at the final iteration. We use the following formula to calculate the size of the population for iteration $N_{G+1}$:

20 $$\begin{aligned} \begin{aligned} N_{G+1}=round(N_{init}(1-\dfrac{N_{init}-N_{min}}{nfes_{max} }))_G, \end{aligned} \end{aligned}$$

where $N_{min}=4$, $nfes_{max}$ is the maximum number of function evaluations allowed. Whenever $N_{G+1}<n_G$, we remove the $(N_G-N_{G+1})$ worst-ranked individual from the population.

The calculation formulas of $l_r$ and $l_c$ are

21 $$\begin{aligned}{} & {} l_{r,g}=\dfrac{\sum _{h=1}^{\left| S_{r,g} \right| }w_{h,g}r_{h,g}^2 }{\sum _{h=1}^{\left| S_{r,g} \right| }w_{h,g}r_{h,g} } \end{aligned}$$

22 $$\begin{aligned}{} & {} l_{c,g}=\dfrac{\sum _{h=1}^{\left| S_{c,g} \right| }w_{h,g}c_{h,g}^2 }{\sum _{h=1}^{\left| S_{c,g} \right| }w_{h,g}c_{h,g} }. \end{aligned}$$

Vectors $S_r$ and $S_c$ denote the rank and c containing successful trials, respectively. $\left| S_{r,g} \right|$ and $\left| S_{c,g} \right|$ represent the lengths of $S_{r,g}$ and $S_{c,g}$, respectively.

**Selection of a new population for the next iteration:**

23 $$\begin{aligned} x_{i,G+1}=\left\{ \begin{aligned} y_{i,G}&,&if f(y_{i,g}) \le f(x_{i,g}) \\ x_{i,G}&,&\mathrm { ~otherwise~}. \end{aligned} \right. \end{aligned}$$

We use greedy selection to update the new population set of the next generation. If the objective function value $f(y_{i,g})$ of the trial solution is not higher than the objective function value $f(x_{i,g})$ of the solution, then $y_i$ replaces $x_i$.

**Fitness function:**

24 $$\begin{aligned} t_j=\sum _{i=1}^{L}\beta _{ig}(DTI_i\times x_j+x_b), \quad j=1,\ldots ,N, \end{aligned}$$

where *L* is the number of hidden layer nodes, $x_i=[x_{i1}, x_{i2},\ldots ,x_{in} ]^T$ is the weight vector that connects the input layer and hidden layer, $x_b=[b_{i1},b_{i2},\ldots ,b_{in} ]^T$ is the bias vector of the hidden layer, and $\beta _i=[\beta _{i1},\beta _{i2},\ldots ,\beta _{im} ]^T$ is the weight vector that connects the hidden layer and output layer. $\beta _i$ can be computed using Eq. (5).

$G(x)=[g(DTI_i,x_1,x_{b1}),g(DTI_i,x_2,x_{b2} ),\ldots ,g(DTI_i,x_n,x_{bn})]$ represents the activation of the hidden layer function:

25 $$\begin{aligned} \begin{aligned} Fitness=\sum _{i=1}^{k} \dfrac{(AUC_i+AUPR_i)}{2}, \quad k=10. \end{aligned} \end{aligned}$$

$AUC_i$ is the area under the receiver operating characteristic (ROC) curve (AUC) obtained using the ELM and $AUPR_i$ is the area under the precision-recall curve (AUCPR) obtained using ELM.

In this study, a DTI pair is input into ELM, that is, drug similarity, protein similarity, and known (or unknown) DTIs are input into ELM, and the predicted new drug–target relationships are output. In SSELM-neg, the connection weight $x_j$ between the input layer and hidden layer, and the bias $x_b$ of the hidden layer are produced using the SS approach, and determine the connection weight between the hidden layer and output layer. The SS approach generates network parameters to enhance the prediction accuracy and generalization ability of the network. In this study, we use 10-fold cross-validation to verify the prediction performance of SSELM-neg.

**Fig. 2:**
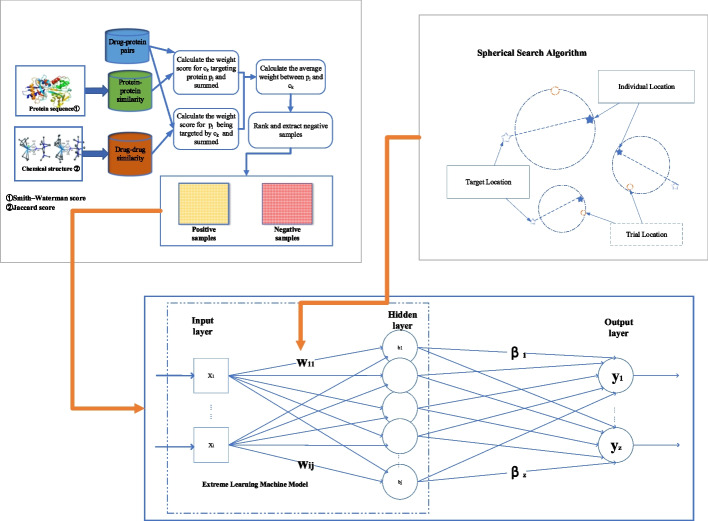
Drug–target interaction prediction framework for SSELM-neg

Our proposed framework is shown in Fig. 2 and the pseudo-code is presented in Algorithm 1.
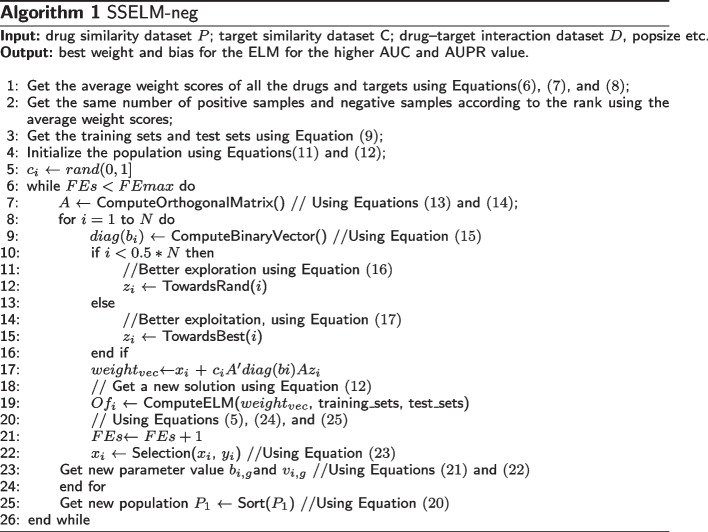

## Experimental evaluation

### Dataset

We compared the performance of our model on the gold standard dataset compiled by Yamanishi [23] with previous excellent methods to demonstrate the effectiveness of our approach. These datasets, derived from databases such as DRUG BANK and Kyoto Encyclopedia of Genes and Genomes 8 (KEGG), correspond to the DTIs of four important protein targets, that is, (i) enzyme (E); (ii) ion channel (IC); (iii) G-protein-coupled receptor (GPCR); and (iv) nuclear receptor (NR), and include 932 drugs, 989 target proteins, and 5,127 mutual relationships between the drugs. In this gold standard dataset, the known DTIs are from multiple public databases, including DrugBank [69], SuperTarget [70], KEGG BRITE [71], and BRENDA [72]. We obtained the similarity between drugs by integrating the chemical structure similarity of the drugs. We downloaded the chemical structures of the drugs from the KEGG LIGAND [71] database, and calculated the similarity using SIMCOMP [73]. We obtained the similarity between proteins by integrating the protein amino acid sequence similarity, which we downloaded from the KEGG GENES database. We obtained the similarity between proteins by integrating the protein amino acid sequence similarity, which we downloaded from the KEGG GENES database [74]. Table 1 presents some statistics for this dataset, including the total number of drugs, total number of targets, and total number of interactions. On average, there are more interactions per drug and target in ICs and Es than in GPCRs and NRs. The details of the gold standard dataset are in Table 1.

After the previous step of establishing a high-confidence negative sample set, we transformed the four interaction datasets into matrix form for the information description: (i) positive interaction and (ii) negative interaction.

**Table 1:** Gold standard dataset

	Interaction	Drug	Target
E	2926	445	664
IC	1476	210	204
GPCR	635	223	95
NR	90	54	26

### Performance evaluation of DTIs

**Fig. 3:**
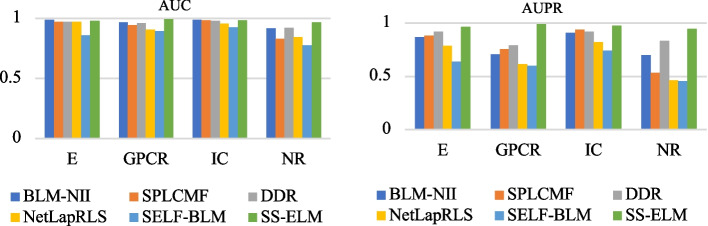
Comparison of the AUC and AUPR results for the six methods

The proposed SSELM-neg model aims to enhance the predictive ability of DTI. In our experiments, we evaluated the predictive capability of the SSELM-neg model on Es, ICs, GPCR, and NRs on the gold standard dataset, and the SSELM-neg model achieved reliable predictive performance. To ensure fairness, we used 10 cross-validation tests to evaluate the performance of SSELM-neg. We divided the gold standard dataset into 10 subsets of equal size. Next, we selected a subset as the test subset to evaluate the prediction results, and used the remaining 9 subsets to train the model. We repeated this process 10 times, each time using a different subset as the test subset. Finally, we obtained the average results from 10 folds. The evaluation metrics are the AUC and area under the precision-recall curve (AUPR). We calculated the ROC curves as shown in Fig. 3 and used AUC as the main quality measure. A precision-recall curve is a graph of the true positive rate (TPR) among all positive predictions for each given recall, and the AUPR value provides a quantitative estimate. The AUPR is suitable for assessing the performance of each method and provides a better estimate of quality because it penalizes the presence of false positives more severely than AUC:

26 $$\begin{aligned}{} & {} TPR=\dfrac{ TP}{TP+FN'} \end{aligned}$$

27 $$\begin{aligned}{} & {} FPR=\dfrac{FP}{TN+FP}'. \end{aligned}$$

The ROC space defines the false positive rate (FPR) as the *x*-axis and the TPR as the *y*-axis. The TPR is the ratio of all samples that are actually positive that were correctly judged as positive. The FPR is the ratio of all samples that are actually negative that were wrongly judged as positive.

### Comparison with other methods

To further illustrate the robustness and effectiveness of the proposed method, we selected four classical methods and four new methods from recent years for comparison: Bigram-PSSM [41], iDTI-ESBoost [75], NRLMF [76], BLM-NII [24], SELF-BLM [77], NetLapRLS [43], SPLCMF [78], and WNN-GIP [79]. To fairly compare DTI prediction performance, we applied these methods to the same gold standard dataset. We also used a randomized setup with 10-fold cross-validation, the same evaluation criteria, and the best parameters for each method. For SSELM-neg, the maximum number of iterations MaxNfes = 10,000, greedy PbestRate = 0.11, population size $PopSize=100$, $rd=0.5$, $c=0.7$, $A_r=1.4$, and historical memory storage size $Ms=5$. The parameters used for the other methods are mentioned in their corresponding articles. For BLM-NII, $g=max$ and $\alpha =0.5$. For SELF-BLM, $c=1$ and $\gamma =1$. For the details of specific parameters, please refer to the original articles.

Table 2 shows the AUC results for each method on the gold standard dataset and Table 3 shows the AUPR results. In these tables, the best results are shown in bold. As shown in Tables 2 and 3, SSELM-neg achieved significantly improved AUC and AUPR performance compared with previous work. The AUPRs for SSELM-neg on E, GPCR, IC, and NR were 0.9652, 0.9906, 0.9762, and 0.9455, respectively, which were higher than those for other advanced algorithms.

Figure 3(left) shows that on GPCR, the AUCs for SSELM-neg were 12%, 6.1%, 2.4%, 2.7%, 5.1%, 4.9%, 8.9%, and 9.9% higher than those for Bigram-PSSM, iDTI-ESBoot, NRLMF, BLM-NII, S PLCMF, WNN-GIP, NetLapRLS, and SELF-BLM, respectively (0.993 vs 0.872, 0.932, 0.969, 0.966, 0.942, 0.944, 0.904, and 0.894, respectively). On NR, the AUCs for SSELM-neg were 10%, 4%, 1.9%, 5.2%, 14.1%, 1.6%, 12.5%, and 19.6% higher than those for Bigram-PSSM, iDTI-ESBoot, NRLMF, BLM-NII, SPLCMF, WNN-GIP, NetLapRLS, and SELF-BLM (0.969 vs 0.869, 0.929, 0.950, 0.917, 0.828, 0.901, 0.844, and 0.773). On IC, the AUCs for SSELM-neg were slightly lower than that for NRLMF (0.988 vs 0.989), but still better than those for the other methods. They were 9.9%, 5.1%, 0.4%, 0.7%, 2.9%, 3.2%, and 6.3% higher than those for Bigram-PSSM, iDTI-ESBoot, BLM-NII, SPLCMF, WNN-GIP, NetLapRLS, and SELF-BLM, respectively (0.988 vs 0.889, 0.937, 0.984, 0.981, 0.959, 0.956, and 0.925, respectively). For Es, our model narrowly outperformed BLM-NII by 0.01% (0.986 vs 0.985), was slightly lower than NRLMF (0.986 vs 0.987), but still far outperformed other models; our model was 3.8%, 1.7%, 1.6%, 2.2%, 1.7%, and 12.6% higher than Bigram-PSSM, iDTI-ESBoot, SPLCMF, WNN-GIP, NetLapRLS, and SELF-BLM, respectively (0.986 vs 0.948, 0.969, 0.970, 0.964, 0.969, and 0.860, respectively). Compared with our model, the state-of-the-art algorithms all had higher AUCs on the E dataset, because it contains the largest number of known DTIs in Es.

Figure 3(right) shows that on the E dataset, the AUPR values for SSELM-neg were 43.6%, 30.2%, 9%, 11.3%, 10.1%, 27.6%, 19.6%, and 34.3% better than those for Bigram-PSSM, iDTI-ESBoot, NRLMF, BLM-NII, SPLCMF, WNN-GIP, NetLapRLS, and SELF-BLM, respectively (0.982 vs 0.546, 0.680, 0.892, 0.869, 0.881, 0.706, 0.786, and 0.639, respectively). On GPCR, the AUPR values for SSELM-neg were 70.9%, 49.1%, 24.2%, 28.2%, 23.7%, 47.1%, 37.4%, and 39.2% higher than those for Bigram-PSSM, iDTI-ESBoot, NRLMF, BLM-NII, SPLCMF, WNN-GIP, NetLapRLS, and SELF-BLM, respectively (0.991 vs 0.282, 0.500, 0.749, 0.709, 0.754, 0.520, 0.617, and 0.599, respectively). On IC, the AUPR values for SSELM-neg were 59.2%, 50.2%, 7.6%, 7.3%, 4.4%, 26.5%, 16.2%, and 23.8% higher than those for Bigram-PSSM, iDTI-ESBoot, NRLMF, BLM-NII, SPLCMF, WNN-GIP, NetLapRLS, and SELF-BLM, respectively (0.982 vs 0.390, 0.480, 0.906, 0.909, 0.938, 0.717, 0.820, 0.744, respectively). On NR, the AUPR values for SSELM-neg were 53.5%, 24.5%, 24.5%, 24.5%, 42.7%, 35.7%, 48.3%, and 48.9% higher than those for Bigram-PSSM, iDTI-ESBoot, NRLMF,BLM-NII, SPLCMF, WNN-GIP, NetLapRLS, and SELF-BLM, respectively (0.946 vs 0.411,0.701, 0.701, 0.701, 0.533, 0.589, 0.463, and 0.457, respectively).

In the four datasets, the average number of interactions between each drug and target was largest in ICs and smallest in NRs. This indicates that the interaction network of ICs contains more information than the interaction network of NRs; hence, the network similarity of ICs is higher and more informative than the network similarity of NRs. NRs contain the largest proportion of ’new drug candidates,’ whereas ICs contain the smallest proportion.

**Table 2:** AUC results for interaction prediction under validation

Method	Datasets			
	E	GPCR	IC	NR
Bigram-PSSM	0.948	0.872	0.889	0.869
iDTI-ESBoost	0.969	0.932	0.937	0.929
NRLMF	**0.987**	0.969	**0.989**	0.950
BLM-NII	0.985	0.966	0.984	0.917
SPLCMF	0.970	0.942	0.981	0.828
WNN-GIP	0.964	0.944	0.959	0.901
NetLapRLS	0.969	0.904	0.956	0.844
SELF-BLM	0.860	0.894	0.925	0.773
SSELM-neg	0.986	**0.993**	0.988	**0.969**

The best results are shown in bold

**Table 3:** AUPR results for interaction prediction under validation

Method	Datasets			
	E	GPCR	IC	NR
Bigram-PSSM	0.546	0.282	0.390	0.411
iDTI-ESBoost	0.680	0.500	0.480	0.701
NRLMF	0.892	0.749	0.906	0.701
BLM-NII	0.869	0.709	0.909	0.701
SPLCMF	0.881	0.754	0.938	0.533
WNN-GIP [79]	0.706	0.520	0.717	0.589
NetLapRLS	0.786	0.617	0.820	0.463
SELF-BLM	0.639	0.599	0.744	0.457
SSELM-neg	**0.982**	**0.991**	**0.982**	**0.946**

The best results are shown in bold

Table 4 shows the results of comparing the AUCs for the different methods using the Friedman test, with our method performing the best. Table 5 shows that there was a significant difference between the performance of the methods.

**Table 4:** Comparison of the AUC results for the methods

Order	Method	Averages rank
1	Bigram-PSSM	2.00
2	iDTI-ESBoost	4.63
3	NRLMF	8.50
4	BLM-NII	6.75
5	SPLCMF	4.75
6	WNN-GIP	4.75
7	NetLapRLS	3.63
8	SELF-BLM	1.50
9	SSELM-neg	8.50

**Table 5:** Friedman test for the AUCs for the methods

Method	Chi-square	Asymptotic significance
Friedman test	27.240	0.001

Table 6 shows the results of the comparison of the AUPRs for the methods using the Friedman test, with our method also performing best. Table 7 shows that there was a significant difference between the performance of the methods.

According to the comparative results of the Friedman test, SSELM-neg was the best (as shown in Tables 4 and 6).

**Table 6:** Comparison of the AUPR results for the methods

Order	Method	Averages rank
1	Bigram-PSSM	1.00
2	iDTI-ESBoost	3.50
3	NRLMF	7.00
4	BLM-NII	6.50
5	SPLCMF	6.75
6	WNN-GIP	3.75
7	NetLapRLS	4.50
8	SELF-BLM	3.00
9	SSELM-neg	9.00

**Table 7:** Friedman test for the AUPRs for the methods

Method	Chi-square	Asymptotic significance
Friedman test	26.555	0.001

### Predicting novel interactions

**Fig. 4:**
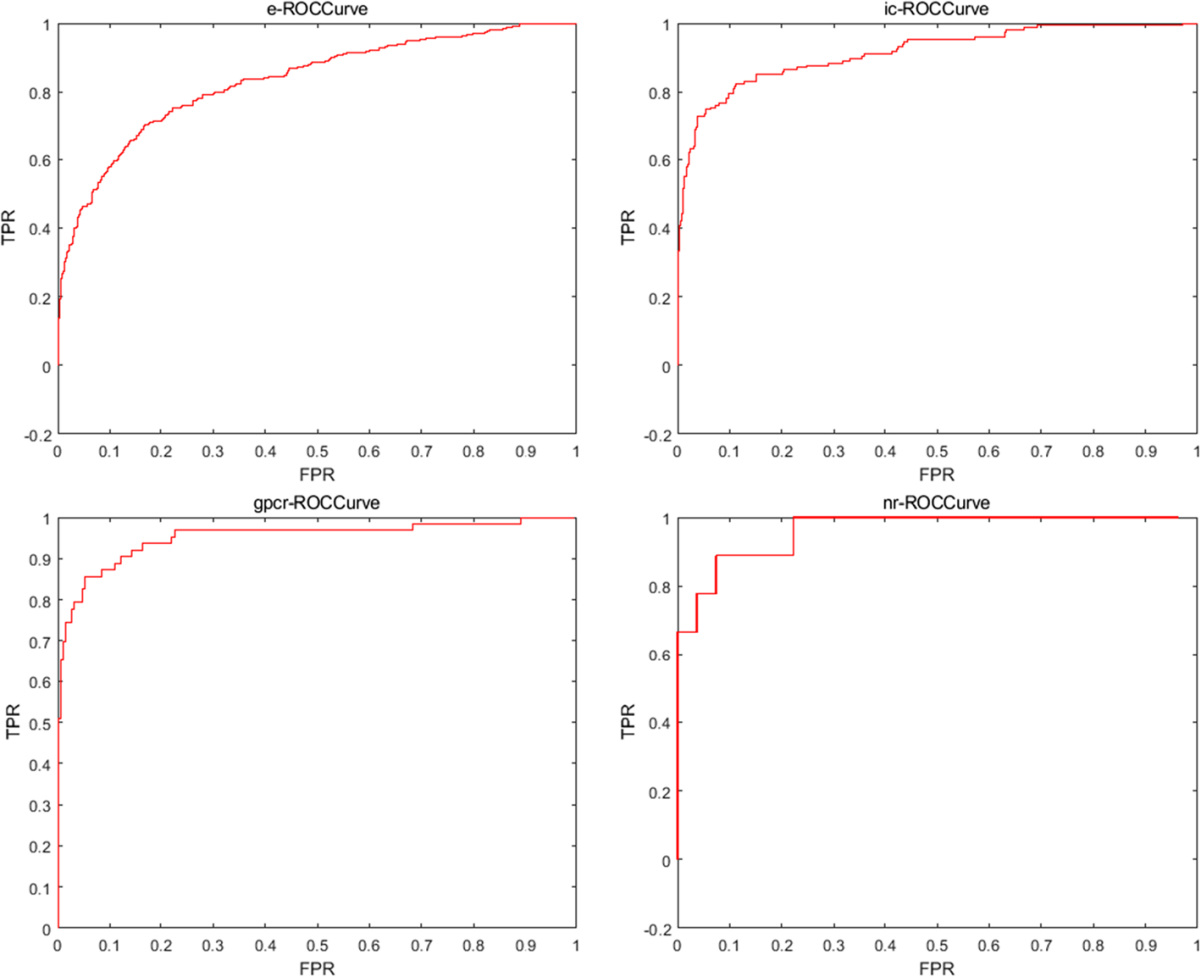
ROC results for interaction prediction for the dataset

To further demonstrate the ability of SSELM-neg to predict a new DTI, we input all the negative samples into SSELM-neg as a test set to predict possible new DTIs. There were no known interactions in the test dataset; hence, we ranked the predicted high DTI scores (possibly positive interactions, but not validated yet) according to their scores, and placed the predicted high scoring interactions in medical biological databases and scientific literature for manual ranking, including DrugBank, KEGG, PubChem, and STITCH. The ROC results of the interaction prediction on the dataset are shown in Fig. 4, the prediction results with interaction after validation are listed in Table 8, and the validation method is marked in the evidence column.

**Table 8:** Prediction results for new drug–target interactions

Drug ID	Drug name	KEGG ID	Target name	Database
D01164	Aripiprazole	hsa3351	5-hydroxytryptamine	DrugBank
			receptor 1B	
D00066	Progesterone	hsa367	Androgen receptor	DrugBank
D00182	Norethindrone	hsa367	Androgen receptor	DrugBank
D00950	Levonorgestrel	hsa367	Androgen receptor	DrugBank
D00954	Norgestrel	hsa367	Androgen receptor	DrugBank
D00294	Diazoxide	hsa8911	Calcium voltage-gated	DrugBank
			Channel subunit alpha1 I	
D00294	Diazoxide	hsa8913	Calcium voltage-gated	DrugBank
			Channel subunit alpha1 G	

The dataset that we used was compiled by Yamanishi. The drug–target interactions contained in the E, IC, GPCR, and NR datasets were extracted from KEGG several years ago, and to allow for a comparison of prediction techniques, they have not been changed [26].

However, with the development of technology, increasing numbers of DTIs have been validated experimentally and their results updated in various biological databases. Therefore, we can compare predicted new interactions in various international public databases. If the predicted new interaction is included in KEGG, DrugBank, or other databases, then we consider the interaction to be valid.

Table 8 shows that our method found many valid interactions, such as Interaction of Aripiprazole (D01164) with 5-hydroxytryptamine receptor 1B(hsa3351); Interaction of Diazoxide with calcium voltage-gated channel subunit alpha1 I; Interaction of Diazoxide with calcium voltage-gated channel subunit alpha1 G; and Progesterone (D00066), Norethindrone (D00182), Levonorgestrel (D00950), and Norgestrel (D00954) all target androgen receptor (hsa367).

The synthetic progestins used to date for contraception and menopausal hormone therapy are derived either from testosterone (19-nortestosterone derivatives) or progesterone (17-OH progesterone derivatives and 19-norprogesterone derivatives). Among the 19-nortestosterone derivatives, the estrane group includes norethisterone and its metabolites, and the gonane group includes levonorgestrel and its derivatives [80]. Aripiprazole (OPC-14597) is a novel atypical antipsychotic drug that is reported to be a high-affinity D2-dopamine receptor partial agonist [81]. It has moderate affinity for the 5-hydroxytryptamine receptor 1B receptor, $6< pKi < 7$ [82].

## Discussion and conclusion

In this study, we proposed a swarm intelligence algorithm-based method for optimizing ELMs called SSELM-neg by integrating drug-drug similarity, protein-protein similarity, and the drug-protein interaction relationship for novel drug-protein interaction predictions. We established a highly credible negative sample dataset, which effectively solved the class imbalance problem between positive and negative samples. We also demonstrated the superior performance of SSELM-neg using results obtained by predicting human DTI networks involving Es, ICs, GPCRs, and NRs.

A small molecule is a type of low molecular weight organic compound with a variety of biological functions. In recent years, mounting evidence has demonstrated the significance of taking microRNAs (miRNAs) as the target of small molecule (SM) drugs for disease treatment [4]. Chen et al. built a computing model of Bounded Nuclear Norm Regularization for SM–miRNA Associations prediction, in which a heterogeneous SM–miRNA network was constructed using miRNA similarity, and a matrix representing the heterogeneous network was defined. Wang et al. [83, 84] proposed a novel method called Dual-Network Collaborative Matrix Factorization for predicting potential SM–miRNA associations [85]. These methods use the similarity matrix of miRNAs, and our method uses the similarity matrix of coding proteins; hence, we believe that it is feasible to improve our method to apply the theory of miRNAs. Drug–target binding affinity prediction is also a research direction for our future work. CHEN et al. proposed a new model called molecular representation block-based drug–target binding affinity prediction (MRBDTA) [86], which showed superior performance in predicting the binding affinity between replication-associated proteins of severe acute respiratory syndrome coronavirus 2 (SARS-CoV-2). In future work, we will focus on predicting the relationship between miRNAs and drugs and on predicting drug target binding affinity.

Machine learning-based methods are used to identify novel DTIs. However, the performance and robustness of this method is data-dependent; hence, inherent knowledge and limited negative samples severely limit the performance of this computational method. In our study, we used drug dissimilarity rules and protein dissimilarity rules to score negative samples, and excluded negative samples with low scores, that is, negative samples that may have interactions between drugs and proteins but have not been verified. Thus, we built a high-confidence and class-balanced train dataset for our SS-ELM model. An ELM is a popular machine learning method that has been widely used in real-world problems because of its fast training speed and good generalization performance. However, in an ELM, randomly assigned input weights and hidden biases often degrade generalization performance. In this study, we assigned input weights and hidden biases using the SS approach to provide the optimized parameters of an ELM. Therefore, it is very suitable to find the optimal network parameters of ELM.

Finally, we input the negative samples that were selected by applying rules to the training set into SSELM-neg. The experimental results verified that our method performed best in terms of identifying DTIs. In the future, we will focus on swarm intelligence optimization for the classifier for the prediction of DTIs.

## Data Availability

The datasets generated and/or analyzed during the study are available at http://web.kuicr.kyoto-u.ac.jp/supp/yoshi/drugtarget/.
